# Changes in Lipids and Lipoprotein Indices in Relation to the Severity of Hypertension in Newly Diagnosed Hypertensive Nigerians

**DOI:** 10.5402/2012/972341

**Published:** 2012-12-11

**Authors:** E. I. Onwubuya, B. C. Anisiuba, C. U. Osuji, J. E. Ahaneku

**Affiliations:** ^1^Department of Medicine, Nnamdi Azikiwe University Teaching Hospital, PMB 5025, Anambra State, Nnewi 435101, Nigeria; ^2^Department of Medicine, University of Nigeria Teaching Hospital, PMB 01129, Enugu State, Enugu 400001, Nigeria; ^3^Department of Chemical Pathology, Nnamdi Azikiwe Teaching Hospital, PMB 5025, Anambra State, Nnewi 435101, Nigeria

## Abstract

Hypertension and dyslipidaemia are important components of metabolic syndrome and both are known to complicate each other. *Materials and Methods*. A total of 149 subjects consisting of 107 hypertensive patients, grouped into 3 (of 37, 35, and 35 patients categorized based on the grade of hypertension as grade 1, grade 2, and grade 3, resp.) and 42 controls, were recruited for this study. Each subject had a recording of the bio- and anthropometric data comprising of the age, height, weight, body mass index (BMI), and abdominal circumference (AC). The blood pressure was also recorded. Fasting blood was collected and serum was used for the estimation of the lipids: total cholesterol (TC), high-density lipoprotein cholesterol (HDL-C), and triglyceride (TG), while low density lipoprotein cholesterol (LDL-C) and VLDL were estimated using Friedewald formula. *Findings*. Patients with hypertension had higher lipid and lipoprotein levels than the controls and the values became more significant with increasing severity of hypertension. The difference was statistically significant for TC, LDL-C, and VLDL-C (*P* < 0.05). *Conclusion*. This study showed that lipid and lipoprotein cholesterol abnormalities exist and even worsen with severity of hypertension. It is important that investigations in patients with hypertension should include a lipid profile.

## 1. Introduction

Hypertension and dyslipidaemia were among the notable risk factors implicated in various cardiovascular events [1–3] both being important modifiable components of metabolic syndrome [4]. Of all the various risk factors for cardiovascular events, the association of hypertension and dyslipidaemia is the commonest [5] though the actual prevalence is not well known.

Various studies have explored the association of hypertension and dyslipidaemia [5–7] but the definite pattern or changes with regard to the severity of hypertension is still largely lacking in reports. Moreover since lipid and lipoprotein indices were common complications of hypertension and/or its treatment [8–10] adequate investigations form the bed-rock of effective patient management [11, 12].

This study was designed to provide the baseline lipid and lipoprotein levels in relation to the severity of hypertension. The knowledge is important because it makes both the clinician and the patient wiser on the lipid risk factor status, in the choice of appropriate antihypertensive medication suitable for the patient, and lastly to recommend strategies aimed at retarding the progression of atherosclerotic process in hypertensive patients. 

## 2. Materials and Methods 

This study was done in the University of Nigeria Teaching Hospital, Enugu, a 760-bed tertiary health institution, designated as Center of Excellence for Cardiovascular diseases in the Southeast, Nigeria. Ethical clearance was obtained from the Hospital's Ethical Review Committee. Subjects for the study were recruited on voluntary basis after due explanation of the purpose.

### 2.1. Selection of Subjects

Patients for the study were recruited from the general outpatient clinic and other clinics of the hospital as they presented for the first time. The control subjects were drawn from the general population of Enugu metropolis. Adult patients aged 15 years and above with established hypertension [13] were recruited, and the controls were normotensive adults. The subjects were matched for age, sex, and BMI.

The exclusion criteria were previous history of hypertension or lipid and lipoprotein abnormalities or on treatment, features suggestive of secondary hypertension, diabetes mellitus, hypothyroidism, nephritic/nephrotic syndrome, chronic liver diseases, patients on steroid therapy, RVD patients on protease inhibitor therapy, current cigarette smoking, regular alcohol use, and for the females recruits, previous history of use of oral contraceptives, pregnancy, and lactation.

A total number of 149 subjects (consisting of both the hypertensive patients and the controls) were recruited for the study [14]. The hypertensive patients (107) were grouped into 3 based on the blood pressure grade levels [15] and the number of hypertensive patients per group was as shown in the following:

 grade 1,37 patients:
 grade 2,35 patients: and
 grade 3,35 patients.

These were matched with the control consisting of 42 normotensive subjects.

### 2.2. Measurements

Blood pressures were recorded according to the guidelines adopted by WHO [13], using a standard Mercury Sphygmomanometer (Acuson brand, UK) with a 23 × 14 cm cuff (bladder 23 × 13 cm). Subjects were seated comfortably in the calm, consulting room with the bare arm resting on a table so that the midpoint of the upper arm was at the level of the heart. They were allowed to relax/rest for about 5 minutes. The cuff of the sphygmomanometer was placed in such a way that the midline of the bladder was over the arterial pulsation and then wrapped and secured snugly around the patients bare upper arm. The lower edge of the cuff was at least 2.5 cm above the antecubital fossa. The cuff was rapidly inflated to 70 mmHg and then by 10 mmHg increment while palpating the radial pulse, noting the reading at which the pulse disappeared and subsequently reappeared during deflation. The bell of the stethoscope was placed over the brachial artery pulsation and the bladder inflated rapidly and steadily to a pressure 30 mmHg above the previously determined level by palpation. This was then gradually deflated at 2 mmHg/second while listening for the appearance of the Korotkoff sounds. The reading at the first appearance of the Korotkoff sound (phase I) was taken as the systolic pressure and when the sound disappeared (phase v) was taken as diastolic pressure. These were recorded to the nearest 2 mmHg of pressure.

Measurements were repeated in 3 different occasions and the average of 3 readings was taken as the systolic and diastolic blood pressures, respectively. Those with systolic blood pressure ≥140 mmHg and/or diastolic blood pressure ≥90 mmHg were taken as hypertensive and graded according to the severity [15] thus:

 grade 1 = 140–159/90–99 mmHg,
 grade 2 = 160–179/100–109 mmHg, and
 grade 3 = >180/>110 mmHg.

#### 2.2.1. Anthropometric Measurements

Height without shoes was recorded in meter (m) and weight without shoes was recorded in kilogram (kg) using a Standiometer (brand UK).

 Body mass index was calculated as weight in kg/(height in m^2^).

(All values were expressed to the nearest one decimal point). Obesity was defined as BMI ≥ 30 Kg/m^2^ [13].

Abdominal circumference was taken as girth around the umbilicus with the subject's body bare and for subjects with pendulous abdomen was taken as midpoint between the lowest part of the rib cage and iliac crest [16]. This was recorded in cm and to the nearest one decimal point. Obesity was defined as abdominal circumference greater than 102 cm (40 inches) for men or greater than 88 cm (37 inches) for women [16].

#### 2.2.2. Lipid and Lipoprotein Measurements

10 mLs of venous blood was drawn from the subjects into a sterile container after overnight or at least 10–12 hours fast [17]. The blood was allowed to clot and the serum was separated from the cells. The serum was stored in deep-freezer until ready for lipid estimation.

Both total cholesterol, HDL-cholesterol and triglycerides were measured by enzymatic method [18, 19]. Measurement of LDL-Cholesterol and very low density lipoprotein (VLDL) required lengthy and elaborate methods that involved use of toxic chemicals and long extraction and clean up procedures with prohibitive cost [17]. Friedewald formula was used in the calculation of these lipoproteins [20] as shown in the following:

 LDL Cholesterol = total serum cholesterol − (HDL + total TG/5*) mg/100 mL
 ∗2.2 if units were in mmol/L
 ∗(VLDL = 1/5 of plasma triglyceride in mg/100 mL).

Lipid and lipoprotein levels were assessed using the monoreagent kit-Diagnostic sera pack testing kit-Quinica Clinica Applicada S.A [QCA] consisting of monoreagent enzymatic cholesterol, cholesterol HDL, and Triglycerides GPO kits. The values were obtained using the colorimetric method (Janeway, England). 

### 2.3. Statistical Analysis

The data were analyzed using the EPI info version 6 and SPSS version 10.0. The variables for each group were presented as mean ± standard deviation. Analysis of variance (ANOVA) was used when there were 3 or more groups and further comparison within groups was carried out using student's *t*-tests. Results were statistically significant at *P* < 0.05. Differences in demographics were assessed using Chi square. Pearson's correlation coefficient was used to determine the association of blood pressures and lipid indices. 

## 3. Results

A total of 107 patients (64 males, 43 females) and 42 controls (21 males, 21 females) were recruited for this study (Table 1).

The mean age for the patients and controls was 44.7 ± 9.5 and 41.4 ± 8.8, respectively, and there was no statistically significant difference (*P* = 0.06) between the patient groups and the control group. Similarly there was no statistically significant difference in the height, weight, body mass index, abdominal circumference, and gender between the patients and the control. However statistically significant difference was observed in the mean values of both the systolic and diastolic pressures between the hypertensive patients and the control group of subjects. Thirty-seven patients (24.8%) had grade 1 hypertension, 35 patients (23.5%) had grade 2 hypertension, and 35 (23.5%) had grade 3 hypertension (Table 2). The mean total cholesterol (Tchol), low density lipoprotein (LDL-C) and very low density lipoprotein (VLDL-C) and also the mean values of the blood pressures were shown in Table 3. The mean total cholesterol (Tchol), low density lipoprotein (LDL-C), and very low density lipoprotein (VLDL-C) in the patients were significantly higher than in the controls. There was no significant difference in their HDL-C and TG levels and in either of the sexes. The mean values of the blood pressures, TC, and VLDL-C increased with the severity of hypertension. Weight of the patients increased with severity of hypertension but only the difference in grade 3 was statistically significant. Age, BMI, and AC also increased with severity of hypertension but the difference was not statistically significant. The changes in the mean values of TG and VLDL-C did not show any consistent pattern in relation to the severity of hypertension.

Of all the variables, only age had a positive correlation (*r* = 0.286, *P* < 0.05) with SBP. Equally age (*r* = 0.197, *P* < 0.05), TC (*r* = 0.334, *P* < 0.05), and LDL-C (*r* = 0.302, *P* < 0.05) had a positive correlation with DBP. In both cases, height was negatively correlated with SBP and DBP (Table 4).

**Figure 1:**
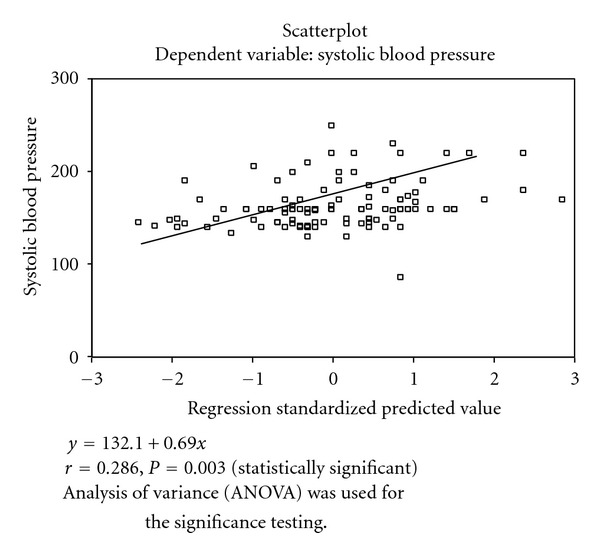
Systolic blood pressure and age.

**Figure 2:**
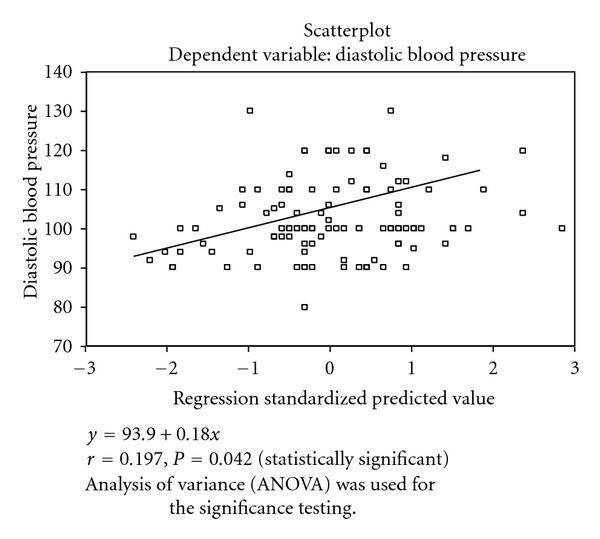
Diastolic blood pressure and age.

**Figure 3:**
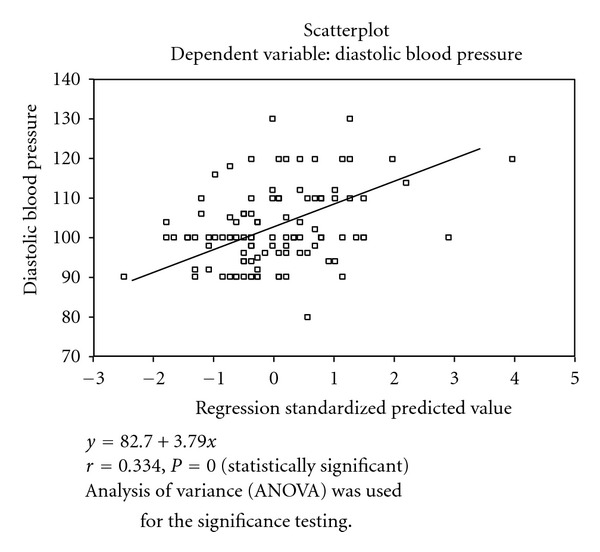
Diastolic blood pressure and total cholesterol.

Figures 1–3 showed the scatter plot for the relationship between DBP, and Age, Tchol, and LDL. These figures showed that DBP in positively correlated with age, Tchol, and LDL. The regression standardized predicted value was *y* = 99.6 + 3.33*x*, where *y* is DBP and *x* is any of the variables (age, Tchol, and LDL).

## 4. Discussion

The result showed that the major differences between the patients and the control group were in the levels of blood pressure as other variables such as age, weight, and BMI were matched. When lipid and lipoprotein indices were compared in the subjects, statistically significant difference was noted in the mean values of TC, LDL-C, and VLDL-C and no difference was observed in the level of HDL. Further analyses of these lipid and lipoprotein indices (ANOVA) in different grades of hypertension showed a statistically significant difference in the levels of VLDL-C in the 3 grades of hypertension while TC and LDL-C were significantly raised in grades 2 and 3 hypertension. There was no statistically significant difference in HDL-C levels when the 3 grades of hypertension were compared with the control values.

Generally the lipid and lipoprotein levels in this study were lower than that of the Caucasians in both the patients and the controls and were even within normal reference range. This is also in keeping with a previous study [21]. Similarly majority of the patients had normal body mass index and abdominal circumference.

This observation in part reflected the source of the patients for the study as majority of the cases were recruited from the GOPD where the bulk of unreferred cases presenting for the first time were seen. The vast majority of the patients seen here belonged to the low socioeconomic or low educational class. The economically more stable class was infrequently seen as they preferred private consultation. Previous studies by Taylor [22], Taylor and Agbedana [23] comparing lipid levels according to socioeconomic or educational level showed that the low socioeconomic or low educational class had lower levels. The differences were attributed to diet and level of physical activity. Similarly Seedat and Mayet [24] demonstrated that in primitive society, people remain thin and lipid levels do not rise with age.

Patients with hypertension may have lipid abnormalities at presentation [25] or following treatment [9], the former reflecting a metabolic syndrome with insulin resistance as a common denominator [4, 16]. This study had recorded a significant increase in lipid and lipoprotein levels in patients as compared with the controls. Further analysis of the difference according to the grades of hypertension showed a high level of VLDL-C in the 3 grades of hypertension while TC and LDL-C were elevated in grades 2 and 3 hypertension. The level of LDL-C was statistically insignificant when compared in the 3 grades of hypertension and the controls. Therefore VLDL-C was the predominant lipoprotein that contributed to the increase in TC observed in grades 2 and 3 hypertensive patients. This was in contrast to other studies where the increase in TC was due to a corresponding increase in the levels of LDL-C and VLDL-C [6]. An explanation of this difference might be that VLDL-C is mainly synthesized in the liver and its triglyceride component is derived from too much fatty acid in circulation and excess dietary carbohydrate [22, 23]. Bruce et al. [26] also explained that excess VLDL-C may further be metabolized through the exchange of their triglyceride for cholesterol ester in LDL-C and HDL-C via the action of cholesterol ester transfer protein. The resulting triglyceride-rich HDL-C particle forms a substrate for hepatic lipase which reduces the size and cause the release of apoprotein A1 which is lost through the kidney. The triglyceride-rich LDL-C particle is hydrolyzed by the endothelial bound lipoprotein lipase and generates small dense LDL-C particle, which was not measured. These lead to a low HDL-C and LDL-C and may account for the observation in this study.

Increase in small dense LDL-C and VLDL-C together with a low HDL-C are known as atherogenic lipid triad and had attracted much interest in most lipid researches [5, 6].

Age is one of the factors that influence the levels of lipid and lipoprotein in a population [27]. This was, however, not apparent in this study as the lipid estimations were not done according to the age group. Earlier population studies [7, 16] showed that cholesterol rises with age with adult levels peaking at 50 years in men and a little later in women. This study recorded no significant difference in both sexes. Grundy [27] noted that adiposity played a significant role in the sex-related changes seen in lipid and lipoprotein indices.

 Of all the dependent variables, only age showed a positive correlation with systolic (*r* = 0.286, *P* = 0.003) and diastolic (*r* = 0.197, *P* = 0.042) blood pressures. Previous studies had noted a rise in blood pressure with age [7] and recently the importance of systolic pressure in the elderly was highlighted [2]. Diastolic blood pressure, in addition, had a strong positive correlation with TC (*r* = 0.334, *P* = 0.000) and LDL (*r* = 0.307, *P* = 0.000). Therefore since other variables were not statistically significant, observed abnormalities in lipid and lipoprotein indices may be contributed solely by the blood pressure (diastolic) since it appeared as the most effective predictor of the abnormalities in lipid and lipoprotein indices in patients with hypertension. Previous experimental studies had shown that elevated blood pressure resulted from increased total content of cholesterol in the arterial intima and in the circulation [28].

In conclusion, this study showed that patients with hypertension had a higher lipid and lipoprotein levels than the controls. The most consistent elevation was seen in levels of TC and VLDL-C. These values became more marked with increasing grades of hypertension. Diastolic blood pressure was the independent variable that predicted elevated Tchol and LDL levels.

It is therefore recommended that in patients with hypertension, determination of the lipid and lipoprotein levels should complement the other investigations. Concerted effort should therefore be made towards early identification of associated risk factors. This is not only important in retardation of disease progression but also in the choice of appropriate medication.

Similar studies in other centers in Nigeria may be advocated and highlighting of the role small dense LDL may further lay credence to the observation in this study.

## 5. Scatter Plots of the Systolic and Diastolic Blood Pressures with Other Variables

The multiple regressions of systolic blood pressure with age are showed in Figure 1.

It showed that systolic blood pressure was positively correlated with age (*r* = 0.286, *P* = 0.003). The regression equation for predicted values is shown as *y* = 132.1 + 0.69*x*.

It showed that SBP has a positive correlation with age, that is, it rises with increasing age.

The multiple regressions of DBP and Age are shown in Figure 2. 

This figure showed that DBP was positively correlated with age (*r* = 0.197, *P* = 0.042). The regression equation for the predicted values is shown as *y* = 93.9 + 0.18*x*.

The multiple regressions of DBP and Tchol are shown in Figure 3.

It showed that DBP had positive correlation with Tchol (*r* = 0.334, *P* = 0.000) with regression equation for predicted value as *y* = 82.7 + 3.79*x*.

## Figures and Tables

**Table 1:** The Demographic and clinical characteristics of the two groups.

Baseline variables	Patients	Controls	*t* values *df* = 147	*P* values *P* < 0.05
Age (yrs)	44.7 ± 9.5	41.4 ± 8.8	1.95	0.06
Height (cm)	167.6 ± 8.4	168.1 ± 7.2	0.34	0.74
Weight (kg)	73.0 ± 14.2	73.5 ± 11.9	0.20	0.84
Body mass index	26.1 ± 5.0	26.0 ± 4.2	0.11	0.91
Abdominal circumference (cm)	88.8 ± 11.4	89.5 ± 10.3	0.35	0.73
Systolic blood pressure (mmHg)	163.5 ± 25.4	116.9 ± 9.6	11.54	0.00*
Diastolic blood pressure (mmHg)	102.2 ± 9.7	79.8 ± 10.5	12.41	0.00*
Sex (M/F)	(64/43)	(21/21)	*χ* ^ 2^ = 1.19	0.28

Data presented as mean for each group ± standard deviation.

*Statistically significant*.*

**Table 2:** Gender and grades of hypertension.

Grades of hypertension	Sex		Total (%)
	Male (%)	Female (%)
Grade 1	21	16	37 (24.8)
Grade 2	22	13	35 (23.5)
Grade 3	21	14	35 (23.5)
Controls	21	21	42 (28.2)

Total	85	64	149 (100.0)

*χ* ^2^ = 0.48; *P* = 0.49, *df* = 3.

No statistically significant difference in the sex distribution of the subjects.

**Table 3:** Clinical and laboratory characteristics of the subjects according to the grades of hypertension.

Baseline variables	Hypertensives			Controls (*N* = 42)
	Grade 1 (*N* = 37)	Grade 2 (*N* = 35)	Grade 3 (*N* = 35)
Age (years)	40.2 ± 9.9 (*P* = 0.46)	43.3 ± 9.6 (*P* = 0.59)	48.7 ± 9.9 (*P* = 0.47)	41.4 ± 8.8
Height (cm)	168.1 ± 7.2 (*P* = 0.99)	167.3 ± 9.5 (*P* = 0.09)	167.1 ± 8.6 (*P* = 0.28)	168.1 ± 7.2
Weight (kg)	71.4 ± 11.2 (*P* = 0.72)	71.6 ± 14.1 (*P* = 0.298)	76.2 ± 16.7 (*P* < 0.05)*	73.5 ± 11.9
Body mass index	25.4 ± 4.7 (*P* = 0.484)	25.6 ± 4.8 (*P* = 0.412)	27.2 ± 5.5 (*P* = 0.099)	26.0 ± 4.2
Abdominal circumference (cm)	86.7 ± 10.0 (*P* = 0.861)	88.5 ± 11.5 (*P* = 0.497)	91.4 ± 12.5 (*P* = 0.236)	89.5 ± 10.3
Systolic blood pressure (mmHg)	143.7 ± 6.7 (*P* < 0.05)*	159.8 ± 14.7 (*P* < 0.05)*	187.9 ± 26.2 (*P* < 0.05)*	116.9 ± 9.6
Diastolic blood pressure (mmHg)	93.1 ± 4.2 (*P* < 0.05)*	101.3 ± 3.1 (*P* < 0.05)*	112.7 ± 7.9 (*P* < 0.05)*	79.8 ± 10.4
TC (mmol/dL)	4.98 ± 0.7 (*P* = 0.339)	4.99 ± 0.9 (*P* < 0.05)*	5.43 ± 0.9 (*P* < 0.05)*	4.75 ± 0.6
HDL-C (mmol/dL)	1.33 ± 0.4 (*P* = 0.076)	1.33 ± 0.4 (*P* = 0.079)	1.33 ± 0.4 (*P* = 0.079)	1.29 ± 0.3
LDL-C (mmol/dL)	2.99 ± 0.7 (*P* = 0.339)	3.09 ± 0.9 (*P* = 0.012)	3.51 ± 0.9 (*P* = 0.014)	2.87 ± 0.6
VLDL-C (mmol/dL)	0.62 ± 0.2 (*P* < 0.05)*	0.54 ± 0.2 (*P* < 0.05)*	0.58 ± 0.2(*P*, 0.05)*	0.51 ± 0.1
TG (mmol/dL)	1.31 ± 0.6 (*P* < 0.05)*	1.15 ± 0.4 (*P* = 0.079)	1.25 ± 0.3 (*P* = 0.992)	1.09 ± 0.3

Data are presented as mean for each group ± standard deviation.

Analysis of variance (ANOVA) was used for the significance testing.

*Statistically significant difference from the controls.

**Table 4:** Correlation between SBP and DBP with other variables.

Baseline variables	Correlation coefficient (SBP) (*P* values)	Correlation coefficient (DBP) (*P* values)
Age (yrs)	0.286 (*P* < 0.05)	0.197 (*P* < 0.05)
Height (cm)	−0.037 (*P* = 0.702)	−0.054 (*P* = 0.582)
Weight (kg)	0.009 (*P* = 0.929)	0.117 (*P* = 0.229)
Body mass index	0.009 (*P* = 0.927)	0.149 (*P* = 0.125)
Abdominal circumference (cm)	0.019 (*P* = 0.842)	0.125 (*P* = 0.200)
TC (mmol/dL)	0.159 (*P* = 0.101)	0.334 (*P* < 0.05)*
HDL-C (mmol/dL)	0.013 (*P* = 0.894)	0.001 (*P* = 0.992)
LDL-C (mmol/dL)	0.178 (*P* = 0.067)	0.307 (*P* < 0.05)*
VLDL-C (mmol/dL)	−0.096 (*P* = 0.326)	0.010 (*P* = 0.918)
TG (mmol/dL)	0.049 (*P* = 0.615)	0.049 (*P* = 0.615)

*Correlation is significant.
